# Activation of PI3Kγ/Akt pathway increases cardiomyocyte HMGB1 expression in diabetic environment

**DOI:** 10.18632/oncotarget.13096

**Published:** 2016-11-04

**Authors:** Jia Song, Qian Liu, Han Tang, Aibin Tao, Hao Wang, Raymond Kao, Tao Rui

**Affiliations:** ^1^ Division of Cardiology, Department of Medicine, The Affiliated People's Hospital of Jiangsu University, Zhenjiang, Jiangsu, 212002, China; ^2^ Critical Illness Research, Lawson Health Research Institute, London, Ontario, N6A 4G5, Canada; ^3^ Departments of Medicine, Pathology and Laboratory Medicine, Schulich School of Medicine and Dentistry, Western University, London, Ontario, N6A 4G5, Canada

**Keywords:** cardiomyocyte, HMGB1, PI3Kγ, Akt, diabetes mellitus

## Abstract

**Background:**

The high mobility group box 1 (HMGB1) protein mediates the cardiomyocyte–cardiac fibroblast interaction that contributes to induction of myocardial fibrosis in diabetes mellitus (DM). In the present study, we aim to investigate the intracellular signaling pathway that leads to cardiomyocyte HMGB1 expression under a diabetic environment.

**Results:**

HMGB1 expression is increased in high concentration of glucose (HG)-conditioned cardiomyocytes. Challenging cardiomyocytes with HG also increased PI3Kγ and Akt phosphorylation. Inhibition of PI3Kγ (CRISPR/Cas9 knockout plasmid or AS605240) prevented HG-induced Akt phosphorylation and HMGB1 expression by the cardiomyocytes. In addition, inhibition of Akt (Akt1/2/3 siRNA or A6730) attenuated HG-induced HMGB1 production. Finally, challenging cardiomyocytes with HG resulted in increased reactive oxygen species (ROS) production. Treatment of cardiomyocytes with an antioxidant (Mitotempo) abolished HG-induced PI3Kγ and Akt activation, as well as HMGB1 production.

**Materials and Methods:**

Isolated rat cardiomyocytes were cultured with a high concentration of glucose. Cardiomyocyte phosphatidylinositol 3-kinase gamma (PI3Kγ) and Akt activation were determined by Western blot. Cardiomyocyte HMGB1 production was evaluated with Western blot and enzyme-linked immunosorbent assay (ELISA), while cardiomyocyte oxidative stress was determined with a DCFDA fluorescence probe.

**Conclusions:**

Our results suggest that the cardiomyocytes incur an oxidative stress under diabetic condition, which subsequently activates the PI3Kγ/Akt cell-signaling pathway and further increases HMGB1 expression.

## INTRODUCTION

Diabetes mellitus (DM) is a chronic metabolic disease. The number of people with diabetes has increased to close to 400 million worldwide. It is recognized as a globe health burden [1, 2]. As the number of diabetic patients increases, a worldwide epidemic of diabetic complications has followed. Myocardial dysfunction is one of the major complications of DM [3]. Changes in myocardial structure and function occur in DM, which ultimately result in ventricular dysfunction and heart failure [4, 5]. However, the molecular mechanisms underlying myocardial dysfunction in DM remain unclear.

The heart is an organ composed of several different cells [6]. Among the myocardial cells, cardiomyocytes represent the most important functional cell, and cardiac fibroblasts constitute one of the most abundant non-cardiomyocytes. Both cardiomyocytes and fibroblasts are important for normal cardiac function [7]. In pathological conditions, myocardial dysfunction can be induced by faulty cell–cell communication between cardiomyocytes and other heart cells (e.g., cardiac fibroblasts) [8, 9]. Our previous study demonstrated that in diabetic condition, cardiomyocytes increase the expression of the high-mobility group box 1 (HMGB1) protein, which mediates cardiomyocyte–fibroblast communication, thus resulting in fibroblast collagen production and myocardial fibrosis [10]. However, the intracellular signaling pathway involved in the regulation of HMGB1 expression by the cardiomyocytes needs to be determined.

Phosphatidylinositol 3-kinases (PI3K) are a family of lipid kinases that produce phosphatidylinositol 3,4,5-triphosphate, which provides a membrane docking site for the serine/threonine kinase protein kinase B (PKB), also known as Akt [11]. The p110 catalytic subunit of PI3K has following isoforms namely, α, β, δ, and γ [11]. All of the members of PI3K are expressed in the heart [12]. We have previously reported that PI3Kγ plays a role in the regulation of cardiomyocyte HMGB1 production in sepsis [13]. In the present study, we aim to assess the role of the PI3Kγ/Akt cell-signaling pathway in regulation of cardiomyocyte HMGB1 expression in diabetic condition.

## RESULTS

### Condition of cardiomyocytes with a high concentration of glucose (HG) activates PI3Kγ and Akt; it also increases cardiomyocyte HMGB1 production

**Figure 1:**
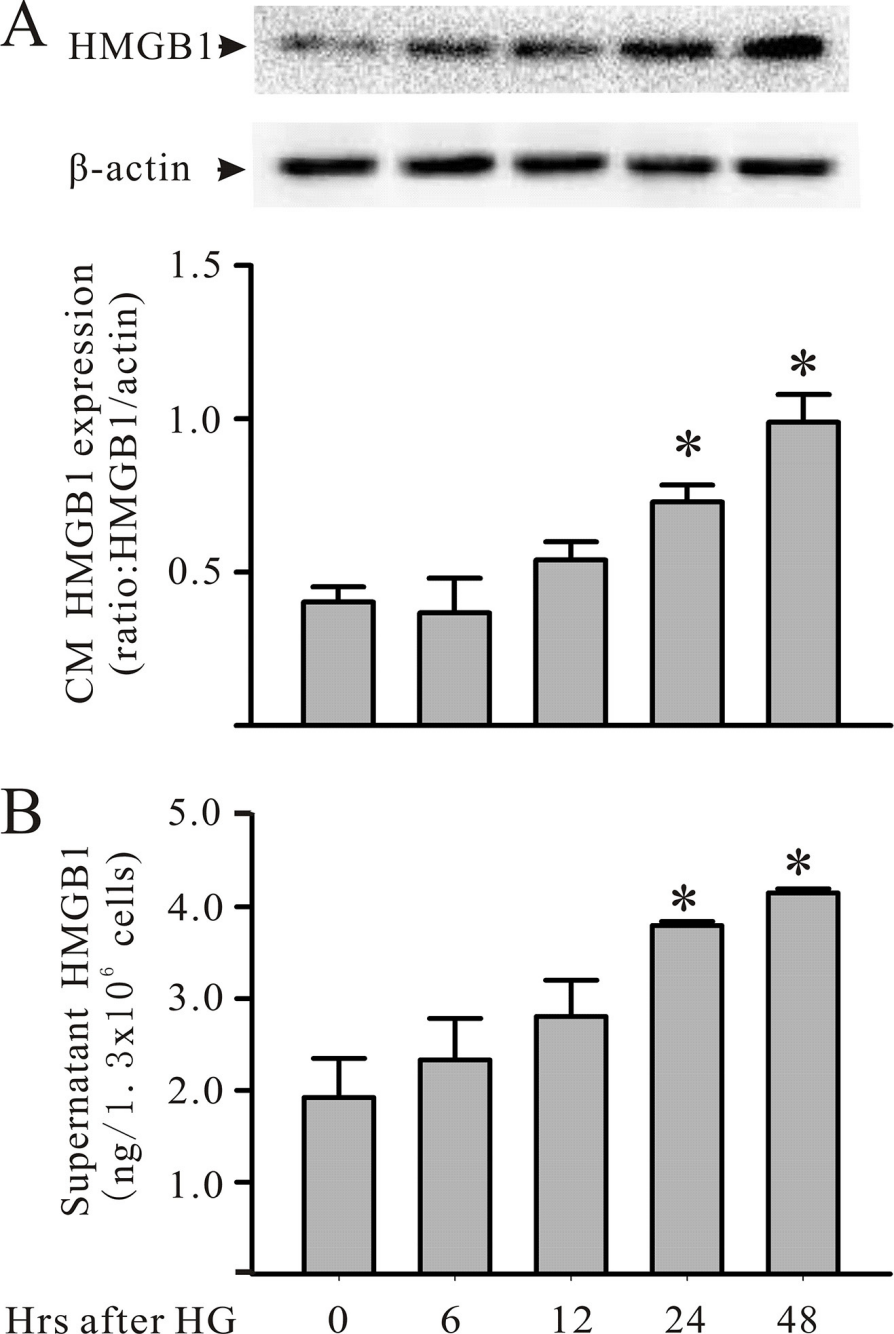
HG increases cardiomyocyte HMGB1 production in culture Mouse cardiomyocytes were exposed to HG (30 mM). Cardiomyocytes and their supernatants were harvested at the indicated times for the assessment of intracellular (**A**: Western blot) and extracellular (**B**: ELISA) HMGB1, respectively. *n* = 3, *P < 0.05 as compared with time 0.

**Figure 2:**
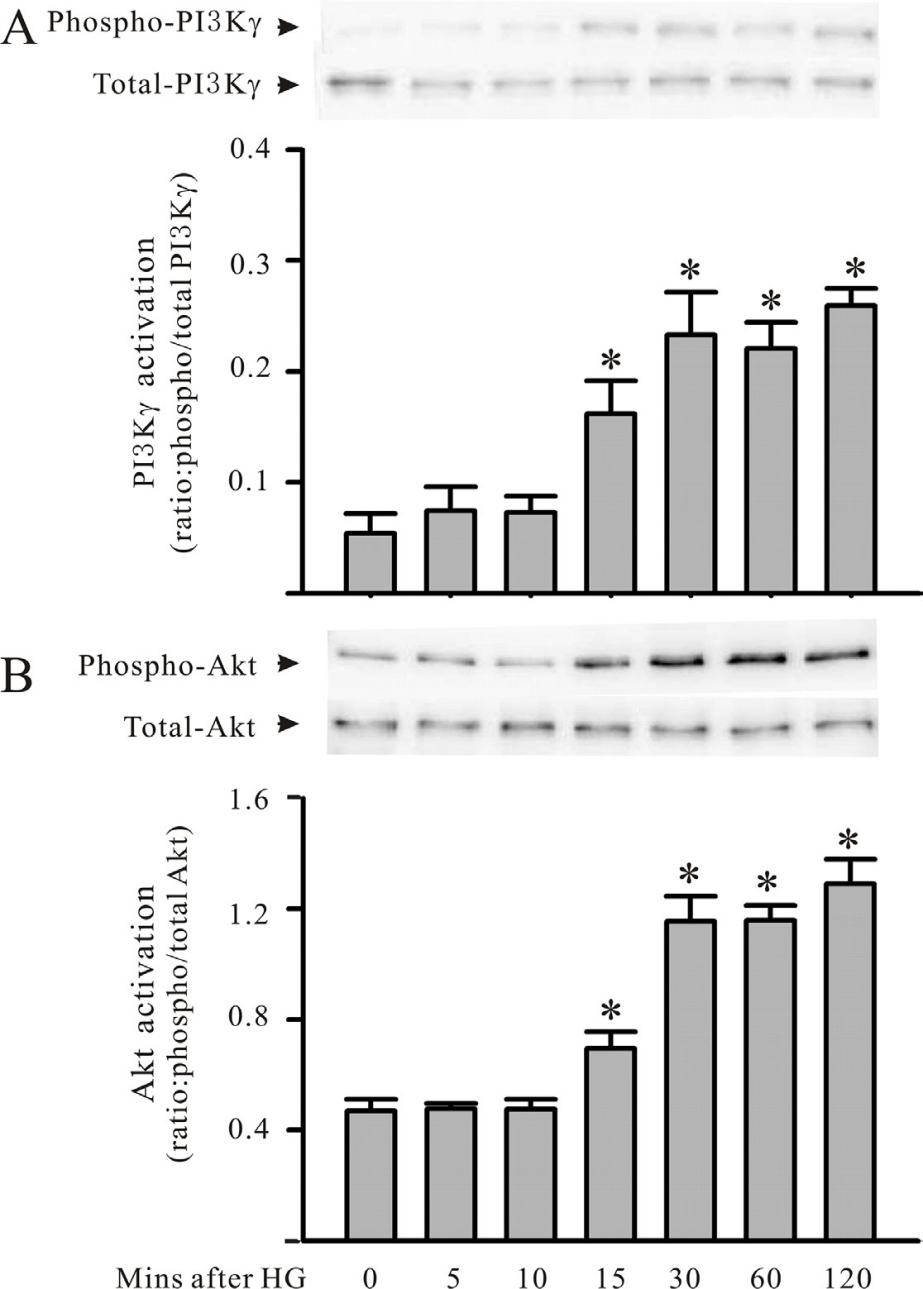
PI3Kγ and Akt are activated in cardiomyocytes under HG condition Cardiomyocytes were treated with HG (30 mM). At the indicated time points, cardiomyocytes were harvested for the assessment (Western blot analysis) of both phosphorylated and total PI3Kγ (**A**) and Akt (**B**) proteins. Representative blots are shown above, and densitometric analysis is visible below. *n* = 3, *P < 0.05 when compared with control (time 0).

Cardiomyocytes were cultured with HG for up to 48 hours. The cardiomyocytes or supernatants were harvested to assess HMGB1 expression/production for the protein phosphorylation assay. As shown in Figure 1, challenging the cardiomyocytes with HG increased HMGB1 expression and secretion. In addition, challenging the cardiomyocytes with HG also resulted in the activation of both PI3Kγ and Akt, as indicated by the increase in PI3Kγ and Akt phosphorylation of the cardiomyocytes (Figure 2). Both PI3Kγ and Akt phosphorylation increased at 15 minutes, and peak activation was noted 30 minutes after HG treatment.

### The PI3Kγ/Akt pathway regulates cardiomyocyte HMGB1 expression

**Figure 3:**
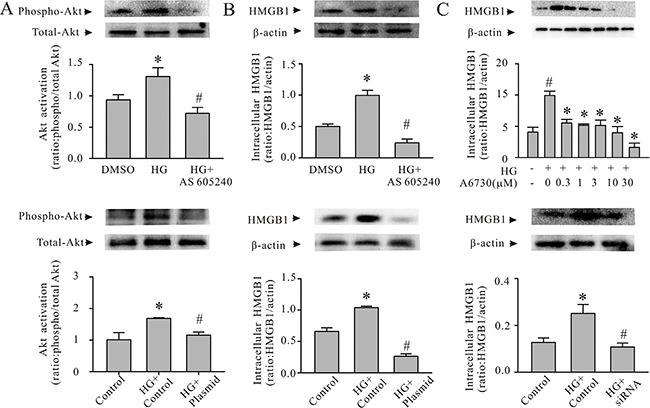
The PI3Kγ/Akt pathway is associated with the expression of HMGB1 in HG-conditioned cardiomyocytes A and B: Cardiomyocytes were pretreated with either AS605240 (a PI3Kγ inhibitor; 0.25 μM) or PI3K p110γ CRISPR/Cas9 KO plasmid. Subsequently, the cardiomyocytes were conditioned with HG or mannitol for either 30 minutes or 24 hours. The cardiomyocytes were harvested for the assessment of Akt phosphorylation (**A**) or HMGB1 expression (**B**). *n* = 3; *P < 0.05 versus dimethyl sulfoxide (DMSO) or control plasmid; ^#^*P* < 0.05 versus HG. (**C)** Upper panel, cardiomyocytes were pretreated with an Akt inhibitor, A6730 (0–30 μM) or DMSO (vehicle) for 1 hour and conditioned with HG for 24 hours. The cardiomyocytes were harvested for the Western blot assessment of HMGB1 production. *n* = 3; *P < 0.05 versus DMSO; ^#^*P* < 0.05 versus HG. Lower panel: cardiomyocytes were transfected with the control or Akt1/2/3 siRNA, and they were then conditioned with HG (30 mM) for 24 hours. The cardiomyocytes were harvested for HMGB1 expression. *n* = 3; *P < 0.05 versus control; ^#^*P* < 0.05 versus HG.

To determine whether the PI3Kγ/Akt cell-signaling pathway regulates HMGB1 expression in cardiomyocytes under diabetic condition, the cardiomyocytes were transfected with PI3K p110γ CRISPR/Cas9 knock-out (KO) plasmid followed by conditioning with HG. As shown in Figure 3A and 3B, knock-down PI3K p110γ in cardiomyocytes prevented Akt phosphorylation and HMGB1 expression after HG challenging. Similarly, pretreatment of cardiomyocytes with the PI3Kγ inhibitor AS 605240 (Cayman Chemical, Ann Arbor, MI, USA) blunted HG-induced Akt activation and abolished the HG-induced increase in cardiomyocyte HMGB1 production (Figure 3A and 3C).

To investigate whether HG-induced HMGB1 expression in cardiomyocytes is dependent on Akt, the cardiomyocytes were either pretreated with an Akt inhibitor (A6730; Sigma-Aldrich, St Louis, MO, USA) or an Akt siRNA, and they were subsequently conditioned with HG. Then, cardiomyocyte HMGB1 expression was determined. As shown in Figure 3C, inhibition of Akt significantly decreased cardiomyocyte HMGB1 expression (Figure 3C). Taken together, these findings indicate that the HG-induced HMGB1 expression in cardiomyocytes is regulated by the PI3Kγ/Akt intracellular signaling pathway.

### Oxidative stress in cardiomyocytes with HG results in PI3Kγ activation

**Figure 4:**
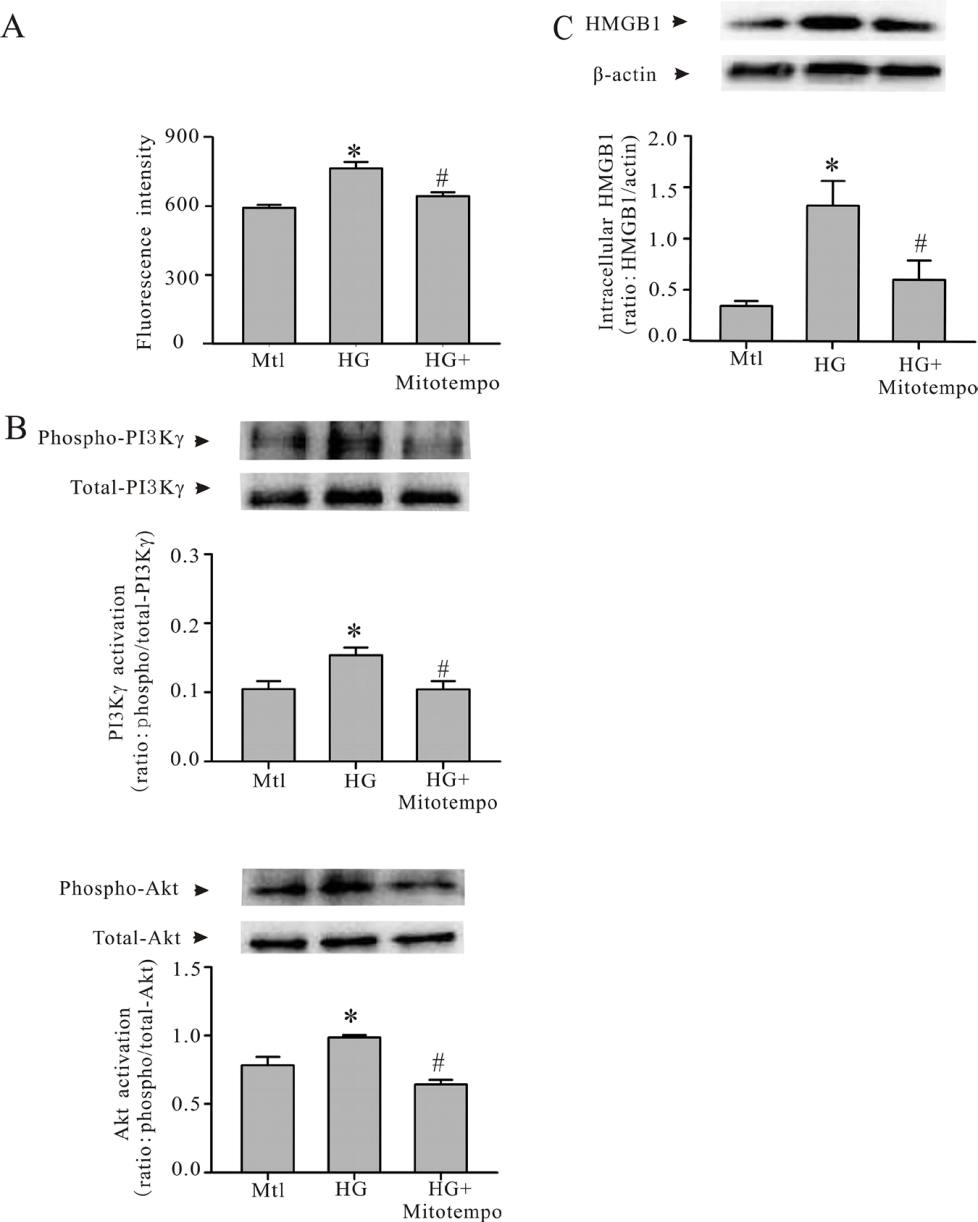
Mitotempo prevents HG-induced PI3Kγ/Akt activation and HMGB1 expression Cardiomyocytes were conditioned with HG or mannitol (Mtl) with or without Mitotempo (25 mM) for 30 minutes or 24 hours. Subsequently, the cardiomyocytes were harvested to detect oxidative stress with a DCFDA microplate assay (**A**); phosphorylation of PI3Kγ and phosphorylation of Akt (**B**); and HMGB1 expression (**C**). *n* = 3, *P < 0.05 compared to Mtl; ^#^*P* < 0.05 compared to HG.

In order to determine whether the condition of cardiomyocytes increased in reactive oxygen species (ROS) generation within the cells, and to assess whether ROS was involved in HG-induced PI3Kγ activation and HMGB1 expression, cardiomyocyte oxidant status was assessed with an intracellular oxidant fluorescence probe, DCFDA. As shown in Figure 4A, the cardiomyocytes incurred oxidative stress, which was attenuated with an antioxidant, Mitotempo (Sigma-Aldrich). In addition, preatreating the cardiomyocytes with Mitotempo prevented the HG-induced activation of PI3Kγ and Akt (Figure 4B), and attenuated HG-induced increase in HMGB1 expression (Figure 4C).

## DISCUSSION

In this study, we demonstrated that challenging cardiomyocytes with HG activates PI3Kγ and Akt, as well as increases HMGB1 expression/production by the cells. Inhibition of PI3Kγ prevents Akt activation and blunts the HG-induced increase in HMGB1 expression by the cardiomyocytes. These results indicated that the PI3Kγ/Akt pathway is pivotal for increasing in cardiomyocytes HMGB1 expression in a diabetic environment. In addition, we demonstrated that cardiomyocytes preconditioned with HG incurred an oxidative stress. Treatment of the cardiomyocytes with an antioxidant prevented the HG-induced activation of the PI3Kγ/Akt pathway and HMGB1 expression. The results suggest that oxidative stress is an upstream event related to activation of the PI3Kγ/Akt pathway in cardiomyocytes following HG. Our study provided a novel intracellular signaling pathway in the regulation of HMGB1 expression by cardiomyocytes under a diabetic environment.

HMGB1 has been reported involved in various pathologies that include ischemia/reperfusion-induced injuries in hepatic cells and myocardial cells, sepsis-induced myocardial dysfunction, acute lung inflammation, and arthritis [12, 19–21]. It is believed that extracellular HMGB1 functions as a cytokine [14, 15]. HMGB1 exerts biological roles through its interaction with toll-like receptor 4 (TLR4) and receptor for advanced glycation end-products (RAGE) [16]. It binds directly to TLR4 and induces the secretion of proinflammatory cytokines in macrophages and dendritic cells [16]. We previously demonstrated that HMGB1 interacts with the TLR4 of cardiac fibroblasts, which mediates cardiomyocyte–fibroblast interactions under diabetic condition. An increase in HMGB1 promoted the cardiomyocyte-dependent induction of myocardial fibrosis [10]. In the present study, our results further support our previous findings, as cardiomyocytes increase HMGB1 expression in diabetic condition.

Since HMGB1 is a nuclear protein and is constitutively expressed by many cells, previous studies have focused on elucidating the mechanisms that control HMGB1 secretion. It has been demonstrated that the release of HMGB1 by inflammatory cells is dependent on PKCα and RAC1 [17, 18]. The release of HMGB1 by aging cells is controlled by p53 [19]. Few studies have explored the regulatory cell signaling pathway of HMGB1 expression. Using a mouse model of sepsis, we previously demonstrated that HMGB1 expression in cardiomyocytes is modulated by PI3Kγ [13]. In the present study, we extend our previous findings and observe that in diabetic environment, the HMGB1 expression of cardiomyocytes is regulated by the PI3Kγ/Akt cell-signaling pathway. Interestingly, our finding is consistent with those of a recent *in vivo* study, whereby PI3K inhibition reduces myocardial levels of HMGB1, thus attenuating ischemia-/reperfusion-induced myocardial apoptosis [20].

**Figure 5:**
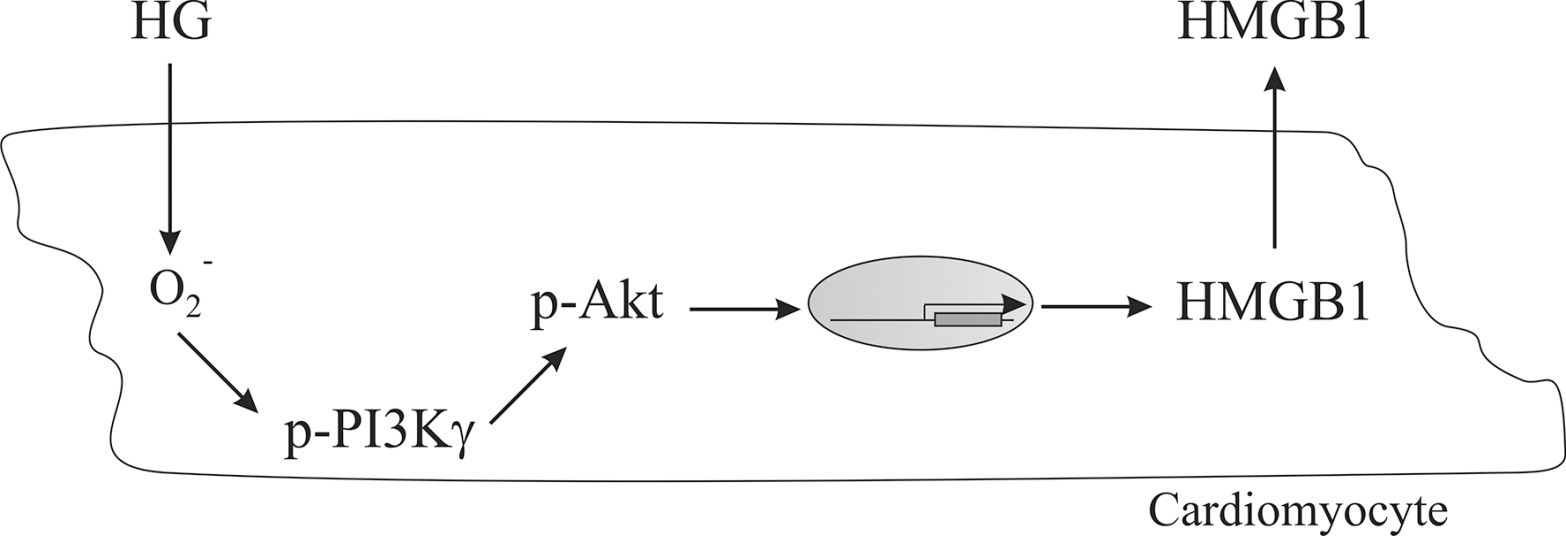
A schematic representation of a working hypothesis illustrating the signaling pathway involved in the induction of HMGB1 expression in cardiomyocytes conditioned with HG

Figure 5 schematically summarizes our working hypothesis on the cell-signaling pathway involved in the induction of HMGB1 expression in cardiomyocytes with HG. The results of our study suggest that under diabetic condition, cardiomyocytes incur oxidative stress and activate the PI3Kγ/Akt-signaling pathway, which further results in HMGB1 production.

## MATERIALS AND METHODS

### Cardiomyocytes

The cardiomyocytes were isolated from the hearts of 1- to 2-day-old neonatal rats, as previously described [10, 21]. Briefly, the harvested hearts were washed with ice-cold Ca++- and Mg++-free Hank's buffer, and they were subsequently minced. The heart tissue was digested with 0.1 Trypsin in Hank's buffer (1:250; Thermo Fisher Scientific, Waltham, MA, USA). The isolated cells were suspended in Dulbecco's Modified Eagle's Medium (DMEM) supplemented with 10% fetal bovine serum (FBS), and they were enriched via a preplating approach to remove contaminating cells before being seeded into cell culture plates. The cardiomyocytes were cultured with DMEM supplemented with 10% FBS for 72 hours; they were then used for the experiments.

### Mimicking a diabetic environment

In order to mimic hyperglycemia *in vivo*, the cardiomyocytes were conditioned with DMEM containing a high concentration of glucose (HG, 30 mM) for up to an additional 48 hours [10, 22]. The control cardiomyocytes were cultured with DMEM containing 30 mM of mannitol (Mtl, osmotic control).

### Cardiomyocyte oxidative stress

To detect the ROS generated in the cardiomyocytes, the cells were seeded in a Fluotrac 96-well flat-bottom cell culture plate (Greiner Bio-One, Frickenhausen, Germany). The cells were challenged with HG for 24 hours. Cardiomyocyte oxidant production was detected with a DCFDA Cellular ROS Detection Assay Kit (ab113851; abcam, Cambridge, MA, USA) according to the manufacturer's instructions. In brief, the cardiomyocytes were washed with 4°C PBS after the cell culture medium was removed, and they were then stained with 100 μL/well of diluted DCFDA solution (final concentration: 20 μM) for 45 minutes at 37°C in the dark. Subsequently, fluorescence intensity was determined with a microplate reader (Spectra MaxGemini, Molecular Devices, Sunnyvale, CA, USA) at excitation and emission wavelengths of 485 nm and 535 nm, respectively.

### Plasmid transfection

The PI3K p110γ CRISPR/Cas9 KO plasmid (cat# sc-422230, Santa Cruz Biotechnology, Dallas, TX, USA) [23] is designed to disrupt gene expression by causing a double-stranded break (DSB) in a 5′ constitutive exon within the *Pik3cg* gene. The cardiomyocytes were transfected with the plasmid using Lipofectamine 2000 reagent (Thermo Fisher Scientific), according to the manufacturer's instructions. The transfection efficiency was ~70%, as determined by Western blot analysis, and the cells were used in experiments 48 hours after the plasmid transfection.

### siRNA transfection

Small-interfering RNA (siRNA) specific for Akt1/2/3 was purchased from Santa Cruz Biotechnology, Dallas, TX, USA (cat# sc-43610). Cardiomyocytes were transfected with the siRNA using Lipofectamine 2000 reagent (Thermo Fisher Scientific) according to the manufacturer's instructions [24]. The transfection efficiency was ~70%, as determined by Western blot analysis, and the cardiomyocytes were used in experiments 48 hours after the siRNA transfection.

### Western blot

Western blot was used to assess intracellular protein phosphorylation and protein expression, as previously described [10]. Briefly, cell lysates were resolved in sodium dodecyl sulfate (SDS)–polyacrylamide gels and transferred to polyvinylidene fluoride (PVDF) membranes. After blocking with 5% nonfat milk, the membranes were immunoblotted with primary antibodies against rat HMGB1 (abcam, Cambridge, MA, USA), Akt (Cell Signaling Technologies, Danvers, MA, USA), p-Akt (Cell Signaling Technologies), PI3Kγ (abcam, Cambridge, MA, USA), p-PI3Kγ (abcam), and β-actin (abcam). Subsequently, the PVDF membranes were incubated with the related secondary antibody, and specific bands were visualized with an ECL detection system and analyzed with the Image J software.

### ELISA

The HMGB1 produced by cardiomyocytes was detected with an HMGB1 enzyme-linked immunosorbent assay (ELISA) detection kit, as previously described [13]. Briefly, the release of HMGB1 by cardiomyocytes was measured by collecting the supernatants and using a two-step sandwich ELISA, according to the manufacturer's instructions.

### Statistical analysis

All data are expressed as the mean ± standard error of the mean (SEM). Statistical analysis was performed using analysis of variance (ANOVA) and Student's *t-test*; a Bonferroni correction was employed for multiple comparisons.
